# Estimating COVID-19 cases infected with the variant alpha (VOC 202012/01): an analysis of screening data in Tokyo, January-March 2021

**DOI:** 10.1186/s12976-021-00146-x

**Published:** 2021-07-17

**Authors:** Hiroaki Murayama, Taishi Kayano, Hiroshi Nishiura

**Affiliations:** 1 School of Medicine, International University of Health and Welfare, Kozunomori 4-3, Narita City, Chiba 286-8686 Japan; 2 Kyoto University School of Public Health, Yoshida-Konoe-cho, Sakyo-ku, Kyoto, 606-8501 Japan

**Keywords:** Statistical estimation, Mutation, Transmissibility, Coronavirus, Epidemiological model, Mathematical model

## Abstract

**Background:**

In Japan, a part of confirmed patients’ samples have been screened for the variant of concern (VOC), including the variant alpha with N501Y mutation. The present study aimed to estimate the actual number of cases with variant alpha and reconstruct the epidemiological dynamics.

**Methods:**

The number of cases with variant alpha out of all PCR confirmed cases was estimated, employing a hypergeometric distribution. An exponential growth model was fitted to the growth data of variant alpha cases over fourteen weeks in Tokyo.

**Results:**

The weekly incidence with variant alpha from 18–24 January 2021 was estimated at 4.2 (95% confidence interval (CI): 0.7, 44.0) cases. The expected incidence in early May ranged from 420–1120 cases per week, and the reproduction number of variant alpha was on the order of 1.5 even under the restriction of contact from January-March, 2021, Tokyo.

**Conclusions:**

The variant alpha was predicted to swiftly dominate COVID-19 cases in Tokyo, and this has actually occurred by May 2021. Devising the proposed method, any country or location can interpret the virological sampling data.

**Supplementary Information:**

The online version contains supplementary material available at 10.1186/s12976-021-00146-x.

## Background

The global pandemic of coronavirus disease (COVID-19), clinically represented by acute infection in upper and/or lower respiratory tract, has established since the emergence of severe respiratory syndrome coronavirus 2 (SARS-Cov-2) in December 2019, Wuhan, China. The widespread epidemiology of COVID-19 is featured by its substantial transmissibility with the estimated basic reproduction number, i.e., the average number of secondary cases generated by a single primary case in a fully susceptible population, ranging from 1.5–3.5 [1]. Once infected, the infection is known to involve greater number of deaths than seasonal influenza with the estimated infection fatality risk ranging from 0.4–3.6% [2, 3]. Due to the absence of specific preventive measures, many industrialized countries have implemented a series of non-pharmaceutical interventions which is in the present day referred to as Public Health and Social Measures, including self-isolation, social distancing, travel restrictions, or lockdown [4–6].

While such interventions have led the paramount impact on social and economic activities in many countries, the variant of concern (VOC) alpha, or the variant that is phylogenetically referred to as B.1.1.7 attracted a global attention [7, 8], rapidly replacing other variants due to 50–70% greater transmissibility and also featured by about 30% greater risk of death than others [9–12]. New and Emerging Respiratory Virus Threats Advisory Group (NERVTAG) in the United Kingdom (UK) identified the common mutation N501Y in the variant alpha [12]. The scientific fact of the increased transmissibility was officially reported as more transmissible in early December 2020, leading to an immediate ban of flights from the UK across the world, but the global spread has been underway, starting to be recognizable especially in European Union countries and the United States [13].

Japan intensified sequencing virus samples from late December both at border quarantine station and domestic testing centers, and also devised a real-time polymerase chain reaction (rt-PCR) technique to detect N501Y mutation as the screening method in each prefecture. VOC and other associated variants, including 501Y.V2 emerging from South Africa (now referred to as variant beta) and 501Y.V3 from Brazil (variant gamma), have then started to be detected in many parts of the country [14]. As of 18 June 2021, totals of 303 cases at the quarantine station and 19,453 domestically acquired cases with variant alpha have been confirmed. Given that only partial samples have been screened out of PCR confirmed cases, a method for estimating actual number of cases with variant alpha was called for. Employing simplistic mathematical models, this study aimed to estimate the actual number of PCR positive cases with variant alpha and reconstruct the epidemiological dynamics so that insights into the current and future prospects can be gained.

## Materials and methods

### Epidemiological data

Weekly number of PCR confirmed COVID-19 cases in Tokyo, January-March 2021 was analyzed. Not only confirmed case count but also numbers of samples screened for N501Y mutation by rt-PCR and positive samples were collected (see Online Supporting Material). The screening was performed via a simple random draw, analyzing viruses of diagnosed cases in an earlier period (e.g. confirmed s = 2 weeks earlier than screening for N501Y). Our analysis was conducted for the dataset from Week 0 to 13, 2021, i.e. from the week starting with 28 December 2020 to that starting with 29 March 2021. The time interval was 7 days throughout the observation period.

### Mathematical model

The data were generated via hypergeometric sampling process. That is, the observed data were considered as resulting from random sampling trials, and the presence of the variant alpha was assumed to follow a hypergeometric distribution. Exploiting the distribution, we would like to understand how many variant alpha cases (*i*_t_) there were in week *t* out of the total of PCR confirmed cases *n*_t-s_ where *s* is the delayed number of weeks for screening (*s* = 2 for the following analyses). Suppose that *m*_t_ samples were screened by rt-PCR in week *t*, the probability of identifying *k*_t_ positive variant alpha cases in the screening samples is

1 $$\mathrm{Pr}\left({X}_{t}={k}_{t};{n}_{t-s},{i}_{t},{m}_{t}\right)=f({k}_{t})=\frac{\left(\begin{array}{c}{i}_{t}\\ {k}_{t}\end{array}\right)\left(\begin{array}{c}{n}_{t-s}-{i}_{t}\\ {m}_{t}-{k}_{t}\end{array}\right)}{\left(\begin{array}{c}{n}_{t-s}\\ {m}_{t}\end{array}\right)}$$

Using Eq. (1) as the likelihood with known datasets *n*_t-s_, *m*_t_ and *k*_t_, we estimated *i*_t_ with the exact 95% confidence interval (CI) derived from the hypergeometric distribution through maximum likelihood method. It should be noted that week *t* in our study represents the week of screening testing for N501Y mutation, not the week of PCR confirmation of cases. To interpret the epidemiological dynamics by the week of diagnosis, two weeks must be subtracted.

Subsequently, assuming an exponential growth for *i*_t_, with the daily growth rate *r*, we consider an exponential growth model *i*(*t*) = *i*_0_exp(*rt*) for continuous time *t* where *i*_0_ is the initial value at *t* = 0. Integrating *i*(*t*) for *Δj* days, i.e., the length of week (i.e., 7 days), we obtain *I*_1_ = *i*_0_(exp(*rΔj*)-1)/*r* for week 0. The same solution for week 1 is *I*_2_ = *i*_0_ exp(*rΔj*) (exp(*rΔj*)-1)/*r*, and we have *I*_3_ = *i*_0_ exp(2*rΔj*) (exp(*rΔj*)-1)/*r* for week 2. Generalizing it, we have E(*i*_t_) = *i*_0_exp(*rwΔj*)(exp(*rΔj*)-1)/*r* for week *w* where *i*_0_ is the initial value in week 0, *r* is the daily growth rate and *Δj* is the length of week. We substituted *i*_t_ in Eq. (1) by the expectation E(*i*_t_) and estimated those unknown parameters *i*_0_ and *r* by minimizing the negative logarithm of the likelihood. The future forecast by Week 18 was obtained with the uncertainty bound calculated from the dataset by Week 13, employing parametric bootstrap method with resampling of parameters *i*_0_ and *r* for 1,000 times.

### Ethical considerations

The datasets used in this study are publicly available, and each of the cases is deidentified before the analysis. Therefore, the present study did not require ethical approval.

## Results

**Fig. 1:**
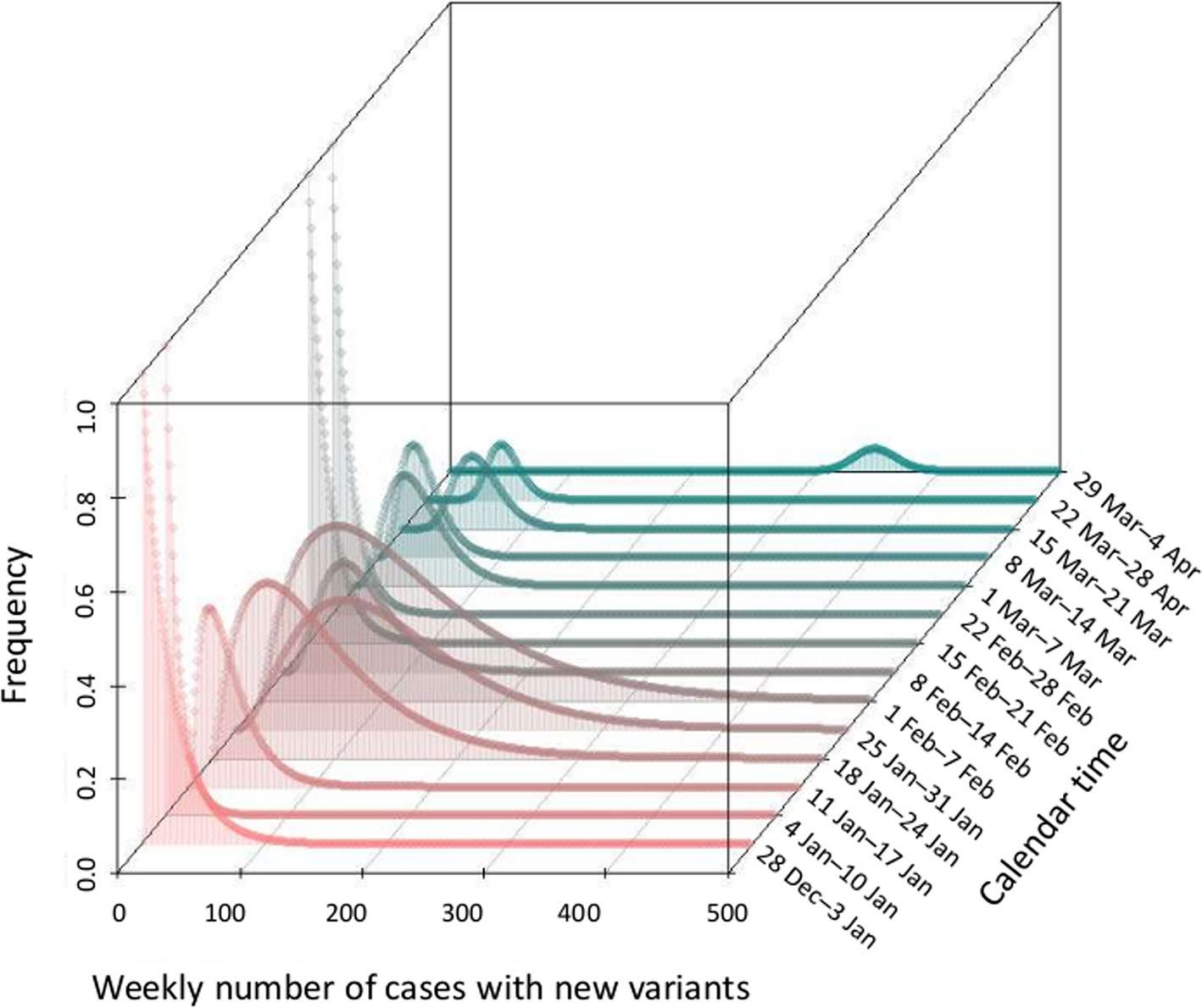
Distribution of estimated cases with the variant alpha per week. The figure shows the estimated distribution of *i*_t_. Only PCR confirmed cases are screened and reflected in the estimate. A hypergeometric distribution was employed to model the data generating process. The 2.5th and 97.5th percentile points of each distribution provide the lower and upper 95% confidence intervals of *i*_t_

Online supporting material shows the empirical data from Week 0 to 13. While no variant was detected from Week 0 to 2, there was 1 positive result each for Week 3 and 4, respectively. These are based on weekly screening testing of 333.6 samples on average, derived from a total of 70, 272 PCR confirmed cases during this period in Tokyo. Namely, from Week 0 to 13, weekly samples of 180, 482, 371, 285, 246, 135, 367, 241, 188, 178, 208, 250, 509, 1030 cases undertook screening. Analyzing the hypergeometric data generating process, the estimated PCR positive variant alpha cases were 0 cases (95% CI: 0, 2) for Week 0 and 1, and 16 cases (95% CI: 1, 89) and 44 cases (95% CI: 2, 243), respectively, for Week 2 and 3 (Fig. 1).

**Fig. 2:**
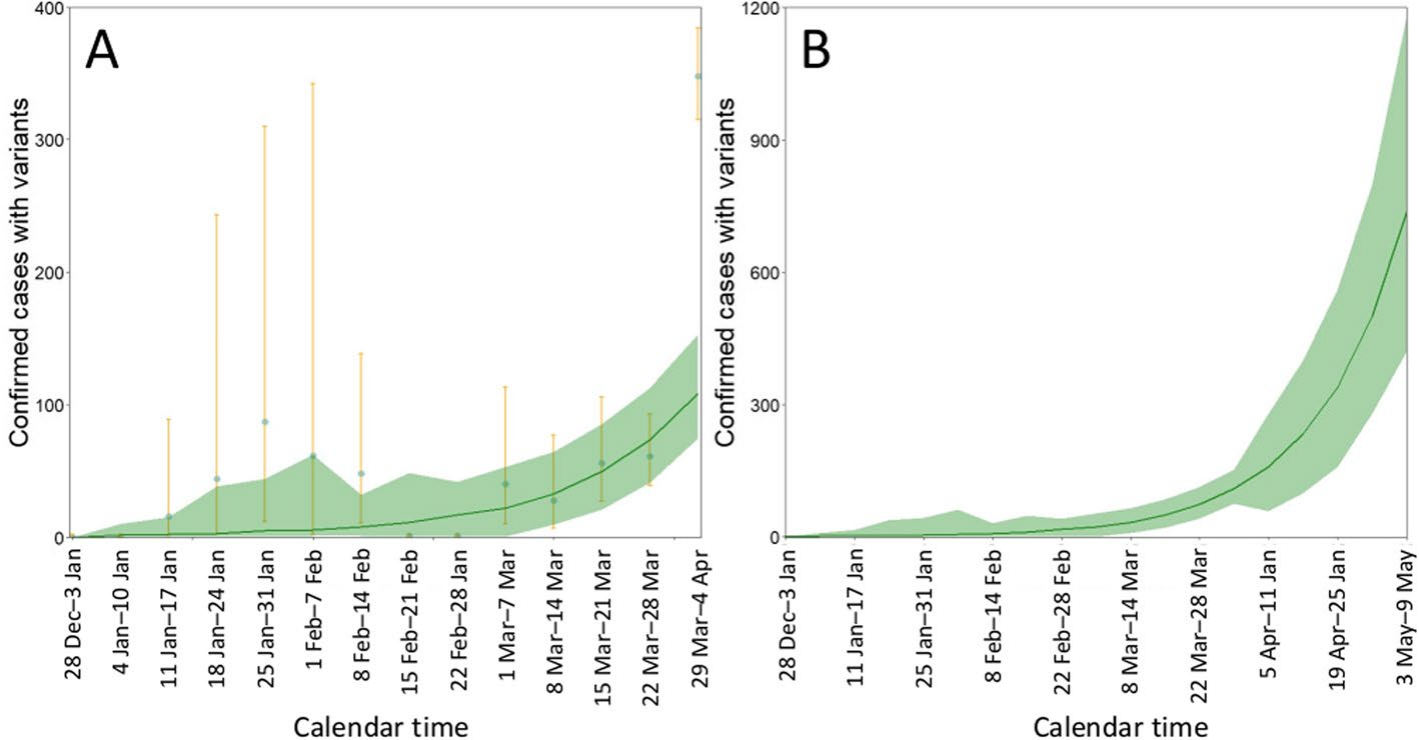
Weekly number of estimated PCR confirmed cases with variant from week 0 to 13 and weekly prediction to week 18. **A** The weekly number of estimated PCR confirmed cases with variant from 28 December 2020 to 29 March 2021. The light blue dot represents sample estimates from empirical data, with the exact uncertainty bound (95% confidence intervals) represented by yellow error bars. The green line shows the simulated mean from the exponential growth model with bootstrap resampling experiments (*n* = 1000 times). The green shade shows the 95% confidence intervals derived from the parametric bootstrap method. **B** The weekly prediction of confirmed cases with variant. The green line shows the simulated mean from the exponential growth model with bootstrap resampling experiments (*n* = 1000 times). The green shade shows the 95% prediction intervals derived from the parametric bootstrap method. The number of PCR testing per week was assumed to be 10,000

Figure 1 purely rests on empirical data alone. However, imposing an exponential growth assumption, the estimated PCR positive variant alpha cases were 0 (95% CI: 0.1, 0.1), 1.8 (95% CI: 0.9, 9.9), 2.5 (95% CI: 0.6, 15.5), 3.1 (95% CI: 0.8, 38.8) and 4.2 (95% CI: 0.7, 44.0) cases, respectively, for Week 0–4 (Fig. 2A). By Week 13, the incidence was estimated to have increased to 107.7 (95% CI: 72.6, 151.3) cases per week.

Parameter *i*_0_ was estimated as 0.09 (95% CI: 0.09, 0.10) and exponential growth rate *r* was estimated to be 0.05 (95% CI: 0.05, 0.06). The latter implies that, assuming that the mean generation time is *T*_g_ = 5 days [15], the reproduction number of variant alpha under the voluntary lockdown period in Tokyo was 1.5 (95% CI: 1.4, 1.5) and 1.6 (95% CI: 1.5, 1.6), respectively, for exponentially distributed and constant generation time (i.e. by using 1 + *rT*_g_ and exp(*rt*) as the estimator). Figure 2B shows the future forecast based on the parameterized model. The expected weekly incidence of variant alpha in Week 18, i.e., from 3–9 May, was 730 cases (95% CI: 420, 1120).

## Discussion

The present study exploited the hypergeometric distribution to estimate the incidence of PCR positive variant alpha cases in Tokyo [12]. Using this simplistic method, we have successfully demonstrated that the estimation task can be simplified and easily integrated into the epidemiological surveillance practice. We have shown that the weekly incidence of variant alpha has at least exceeded 10 cases by mid-February, which was perhaps too late to consider possible elimination, and the estimated reproduction number was on the order of 1.5 even under the restriction of contact. From very limited partial sample data, the present study warned that the variant alpha was expected to swiftly dominate cases soon in Tokyo. Unfortunately, the replacement has actually occurred by May 2021 across Japan.

Devising the proposed method, any other country or location can interpret the partial virological sampling data [11]. Depending on the estimate, stringent countermeasures may be considered, if aiming to fully contain the epidemic and bring the transmission of variant alpha cases under control. To do so, our simplistic method indicates that the numbers of screened samples and positive count (especially, positive cases without an apparent epidemiological link) must be regularly surveyed and reported.

Several limitations must be acknowledged. First, the sample size was very limited in the present study. Especially, empirical observation result during early weeks did not involve any positive screening samples, and exponential growth assumption during the corresponding phase needs to be validated more in the future. At least, we employed the most appropriate statistical inferential approach and attempted to make the best use of the data with uncertainty bound, i.e., the exact and bootstrap-based confidence intervals. Second, our geographic subject was restricted to Tokyo, and we have yet to analyze the dataset in other locations explicitly. In the biggest metropolitan city, we have demonstrated that the variant alpha is perhaps about to replace other strains. Third, the estimated case count is calculated out of all PCR confirmed cases, and there must be greater number of infections with variant alpha.

Despite the abovementioned limitations, we believe that the proposed method successfully established a method for scientifically monitoring the emergence and propagation of the variant alpha. The escaping process of variant alpha cases from entry screening should also be explored to understand the invading dynamic process of the variant.

## Conclusions

The present study offered a method to estimate the actual number of the variant alpha cases out of all PCR confirmed cases. Estimates implied that the variant alpha possesses substantial transmissibility, and have a potential to swiftly dominate COVID-19 cases in Japan.

## Supplementary Information

**Additional file 1.**

## Data Availability

All data generated or analyzed during this study are included in the online supporting material.
